# Effects of cryopreservation on viability and functional stability of an industrially relevant alga

**DOI:** 10.1038/s41598-019-38588-6

**Published:** 2019-02-14

**Authors:** Rahul Vijay Kapoore, María Huete-Ortega, John G. Day, Katarzyna Okurowska, Stephen P. Slocombe, Michele S. Stanley, Seetharaman Vaidyanathan

**Affiliations:** 1 Advanced Biomanufacturing Centre, Department of Chemical and Biological Engineering, The University of Sheffield, Sheffield, S1 3JD UK; 2 Scottish Association for Marine Science, Scottish Marine Institute, Oban, PA37 1QA UK; 3 Department of Plant Sciences, University of Cambridge, Cambridge, CB2 3EA UK

## Abstract

As algal biotechnology develops, there is an increasing requirement to conserve cultures without the cost, time and genetic stability implications of conventional serial transfers, including issues regarding potential loss by failure to regrow, contamination on transfer, mix up and/or errors in the documentation on transfer. Furthermore, it is crucial to ensure both viability and functionality are retained by stored stock-cultures. Low temperature storage, ranging from the use of domestic freezers to storage under liquid nitrogen, is widely being used, but the implication to stability and function rarely investigated. We report for the first time, retention of functionality in the maintenance of master stock-cultures of an industrially relevant, lipid-producing alga, under a variety of cryopreservation regimes. Storage in domestic (−15 °C), or conventional −80 °C freezers was suboptimal, with a rapid reduction in viability observed for samples at −15 °C and a >50% loss of viability, within one month, for samples stored at −80 °C. No reduction in viability occurred at −196 °C. Post-thaw culture functional performance was also influenced by the cryopreservation approach employed. Only samples held at −196 °C responded to nitrogen limitation in terms of growth characteristics and biochemical profiles (lipid production and chlorophyll *a*) comparable to the untreated control, cultured prior to cryopreservation. These results have important implications in microbial biotechnology, especially for those responsible for the conservation of genetic resources.

## Introduction

Biotechnology based on the functional capabilities of microorganisms is hugely important to the global economy. There are numerous products and services derived from microbes contributing to the commercial success of virtually all the major manufacturing sectors including agriculture, bioremediation, biofuels, food, drink, textiles and pharmaceuticals. The economic value of microbial-derived materials is a matter for debate, but healthcare is often highlighted as one of the largest end-user markets for microbial derived products and these had an estimated global value of $100 billion in 2014, increasing to $111 billion in 2015, and are anticipated to exceed $187 billion in value by 2020^1^. To date, only a small proportion of microbial biodiversity has been explored for its commercial potential, with the majority of products and services being derived from a relatively small number of bacteria, including actinomycetes, unicellular yeast and filamentous fungi. Algae are, as yet, relatively unexploited from a biotechnological perspective, but there are an increasing number of companies commercially growing algae for pigment production, health foods, nutraceuticals, cosmetics and cosmeceuticals^2–4^. In addition, aquaculture, specifically the production of early developmental stages of both finfish and molluscs depend on the supply of high quality algae^5^. Furthermore, the high productivity of microalgae, in comparison to terrestrial crops, in conjunction with their high lipid levels have stimulated a great deal of academic and commercial interest in algae for animal food^2,6^, as well as for future biofuels^7^.

Irrespective of their origin and taxonomic classification, biotechnologically exploited microbes and cell-lines are biological resources, working as cell factories producing products or other processes. In algae-based industries, as elsewhere, security of the master stock-cultures, from which the process or product is derived, as well as their functionality (i.e. capacity to continue to produce the metabolite etc. of commercial value) is of paramount importance. If long-term conservation of these cultures in a state where their physical, genetic and functional stability is not possible, then any commercial exploitation will ultimately fail. For microorganisms, in general, a range of options are available to maintain master stock-cultures^8^. These may be categorised under four broad headings namely: (i) conventional serial transfer, most commonly under a cultivation regime that slows metabolic activity; (ii) stored dried, either at room temperature, or a refrigerator at 4 °C; (iii) freeze-drying, or lyophilization; and (iv) cryogenic storage, which may range from employing a domestic freezer to storing in a cryostat under liquid nitrogen at −196 °C.

For algae, serial transfer (i.e. the maintenance of actively growing cultures) has historically been and remains the method of choice for most workers^9^. There are examples of sustained stability, including the ecotoxicology test strain *Chlorella vulgaris* CCAP 211/11B, which was maintained in excess of 40 years under different environmental regimes, with regular transfers to fresh medium, without any detectable genetic or functional changes^10^. However, other algae have been reported to have altered morphologies, growth characteristics and/or have lost the capacity to produce metabolites of commercial interest^11^. Moreover, any maintenance regime that involves routine manipulation increases risks associated with human errors like mislabelling, as well as contamination or culture failure.

Resting spores, or other dormant stages, of some algal species can be maintained dried, stored at ambient or cool temperatures for many years. For example, living aplanospores of the green alga *Haematococcus pluvialis* were recovered from air-dried soil after 27 years storage^12^, and the cyanobacterium *Nostoc commune* was revived from akinetes obtained from dried herbarium specimens after 107 years of storage^13^. Although drying may be applicable for some taxa, the viability levels of dried algae generally decrease with time^14^, and most aquatic algae do not exhibit any persistent dormant stage, making them non-amenable to this approach. Drying has not been widely used and there is no published evidence with respect to retention of functionality or genetic stability of algal cultures stored using this approach. Freeze-drying is the most common technique employed to maintain bacterial and fungal cultures of medical or biotechnological value^15,16^. It has not been found to be a successful method for the conservation of eukaryotic microalgae, with very low levels of viability, <0.001% of the original population, being reported for a range of organisms^17^ and further reductions in viability on prolonged storage^14^. Although, the successful lyophilization of the robust akinete forming cyanobacterium *Nostoc muscorum* has been reported, with no observed reduction in viability after 5 years storage^18^, and this technique has been adopted by a small number of researchers to preserve selected cyanobacterial strains^19^.

Cryopreservation is considered by most Biological Resource Centre (BRC) practitioners to be the optimal long-term conservation method for microbial cultures^8,20^. Where applicable, ultra-low temperature storage permits the maintenance of living organisms in a state of “suspended animation” for considerable periods. In the case of eukaryotic microalgae, no significant reduction in viability has been observed for up to 22 years of cryostorage of a range of taxa^21^. Additionally, the capacity of cells recovered from cryostorage to produce the metabolite(s) of biotechnological interest has also been demonstrated^22–24^. Although at cryogenic storage temperatures all biological functions cease, it is worth noting that upon prolonged storage some chemical damage can still occur due to the free-radical formation and ionising radiation, which may damage nucleic acids and, in time, could influence genetic stability^25^. However, from a practical perspective, viability is considered to be effectively independent of storage duration for decades if not hundreds of years and a half-life for the viability of cryopreserved material has been estimated to be >3000 years^26^. Many bacteria can be successfully cryopreserved with storage at higher sub-zero temperatures, typically in a −80 °C freezer^27^. This technique has also been successfully used for cyanobacteria^19^ but has not been routinely employed for more complex eukaryotic algae. Higher sub-zero storage temperatures in domestic freezers (−15 to −20 °C) are only used for short-term storage, mostly of bacterial cultures, as temperatures above −30 °C generally result in eutectic mixtures where cells are exposed to high salt concentrations^28^.

**Figure 1:**
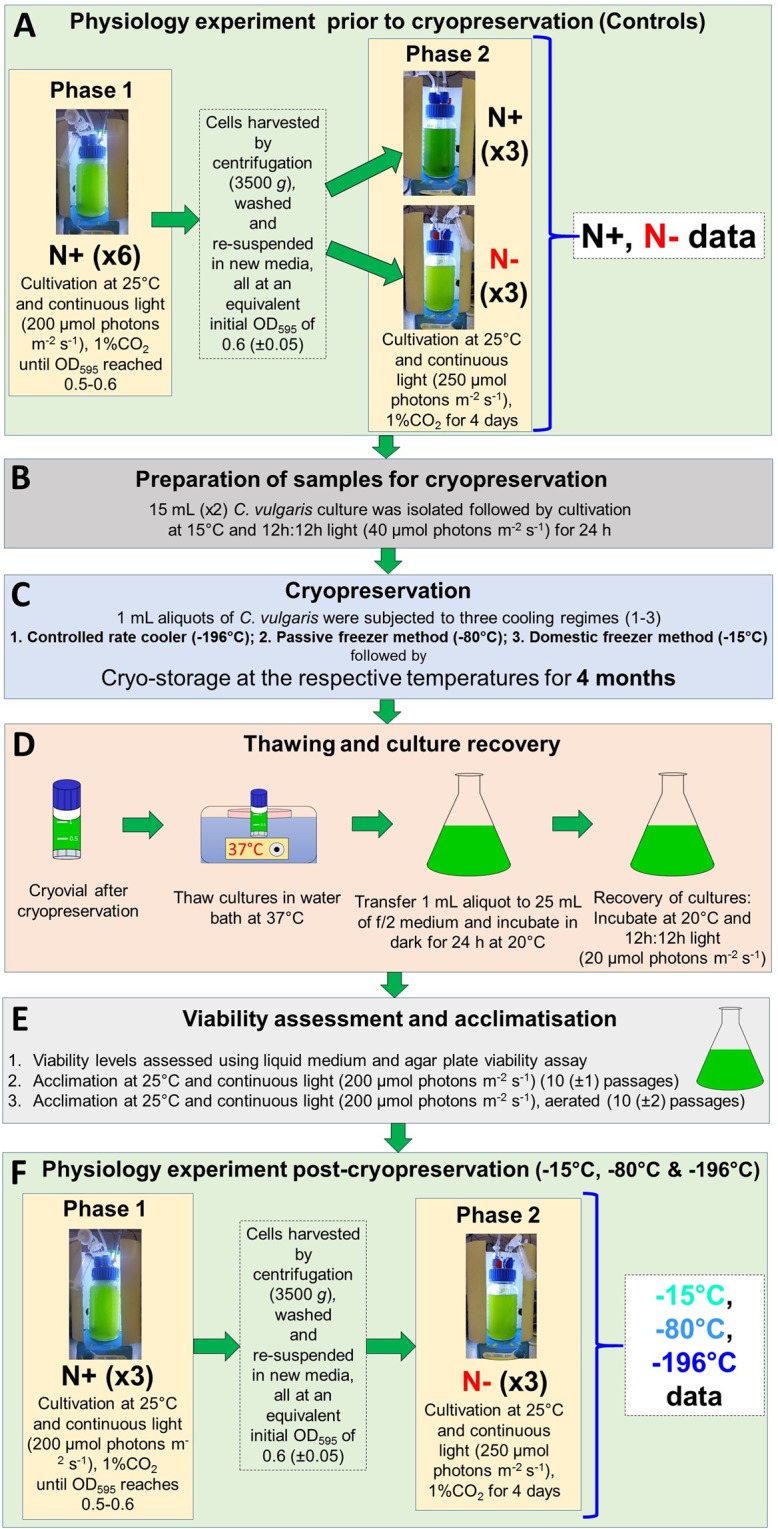
Experimental workflow adopted. The experiment consisted of testing three cryopreservation strategies. This was preceded by a physiological experiment to assess functionality prior to cryopreservation and consisted of growth in two phases (**A**), and subsequent preparation of samples for cryopreservation (**B**). The cryopreservation step (**C**) was then followed by thawing and culture recovery (**D**) and viability assessment and culture acclimatisation (**E**), before repeating the physiological experiment on the cultures post-cryopreservation in two phases (**F**) to test for functionality.

Increasingly non-specialist algal labs, particularly those involved in industrial biotechnology, are looking for approaches suitable for both short-term and long-term preservation. Whilst cryopreservation appears to provide the solution, the guarantee of stability, which is widely highlighted as the strength of the approach, has not been systematically demonstrated in the context of post-thaw functionality. In this study, the post-cryopreservation recovery, growth and biochemical functionality of the biotechnologically relevant alga *C. vulgaris* CCAP 211/21A, which is known to produce up to 52% lipid on a dry weight basis^29^, were studied. As relatively few labs have access to ultra-low temperature refrigerators or liquid nitrogen storage dewars, but most have easy access to domestic or −80 °C freezers, the study was expanded to explore whether these provide a valid option for storage (Fig. 1). The study aims to provide key evidence-base to inform best practice in the biotechnology sector concerning biosecurity of the genetic resources, in turn consolidating the foundation on which the sector is being built.

## Results

### Effects of cooling regime and storage on *C. vulgaris* viability

A graphical description of the experimental workflow can be found in Fig. 1, including details of the cryopreservation treatments and the physiological experiment carried out to test functionality. Simple re-growth assay of the cultures was unaffected by duration of storage up to 4 months at −80 °C and −196 °C (Table 1). However, re-growth of cultures was impacted at −15 °C after only 1 month of storage. This indicated that −15 °C approach was fundamentally unreliable. In terms of absolute viability levels, cells held at −196 °C maintained near 100% viability for 4 months. In contrast, significant reductions (from 98% to 28%) were seen after 1 and 4 months at −80 °C (suggesting a half-life of 1–2 months) and viability was lost after 1 month at −15 °C as judged by the pour-plate assay. Therefore, a closer analysis at the cellular level suggested that there were long-term limitations in the use of the ‘lab-freezer’ approaches, favouring the cryopreservation approach.

**Table 1:** Viability levels of *C. vulgaris* CCAP 211/21A after storage.

Duration of storage	Storage temperature	Capacity of stored algae to re-grow^a^	Viability level^b^
24 h	−15 °C	+++	65 ± 12
	−80 °C	+++	98 ± 3
	−196 °C	+++	97 ± 6
1 month	−15 °C	+−−	<1^*^
	−80 °C	+++	44 ± 21
	−196 °C	+++	97 ± 9
4 months	−15 °C	++−	<1^*^
	−80 °C	+++	28 ± 6
	−196 °C	+++	97 ± 10

^a^Triplicate samples tested in liquid medium: (+) indicates growth and regeneration of an apparently normal culture in an individual replicate; (−) indicates no recovery observed within 4 weeks.

^b^Viability levels (colony formation in agar) expressed as a percentage of the original cell density.

^*^No growth observed in agar plate viability assay, level estimated on basis of regrowth in logarithmic dilutions of the sample after rewarming.

### Post-storage performance, functional phenotype of *C. vulgaris*

**Figure 2:**
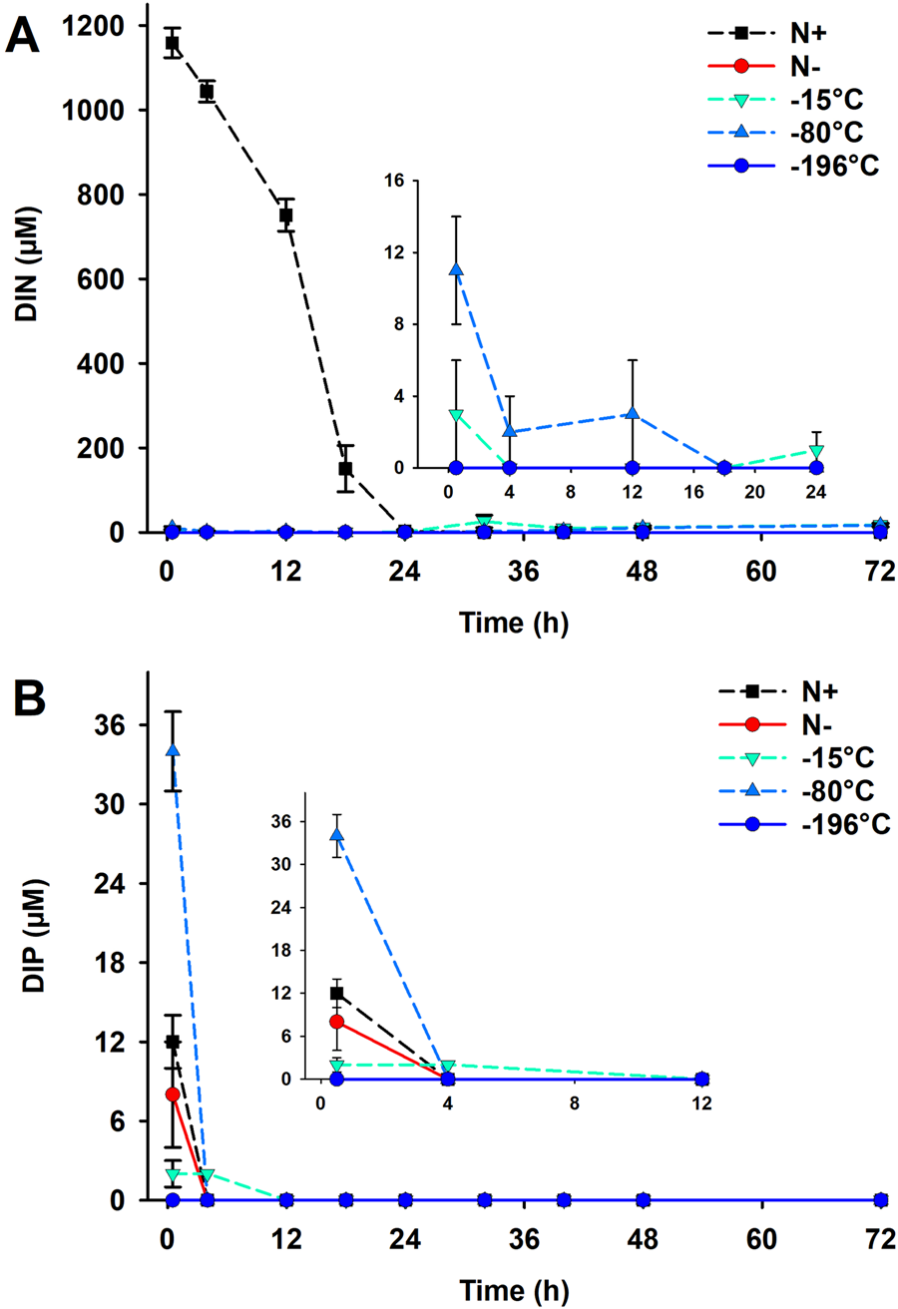
Changes in DIN (**A**) and DIP (**B**) in the media during Phase 2 of the physiological experiment. Mean (±standard error) values of three biological replicates are plotted. The inset in (**A**) shows the changes in DIN for all the N- conditions for 0–24 h, whilst the inset in (**B**) shows changes in DIP for all the conditions in the initial 12 h. 0 h in practice was 30 min (0.5 h) after initiation of Phase 2.

**Figure 3:**
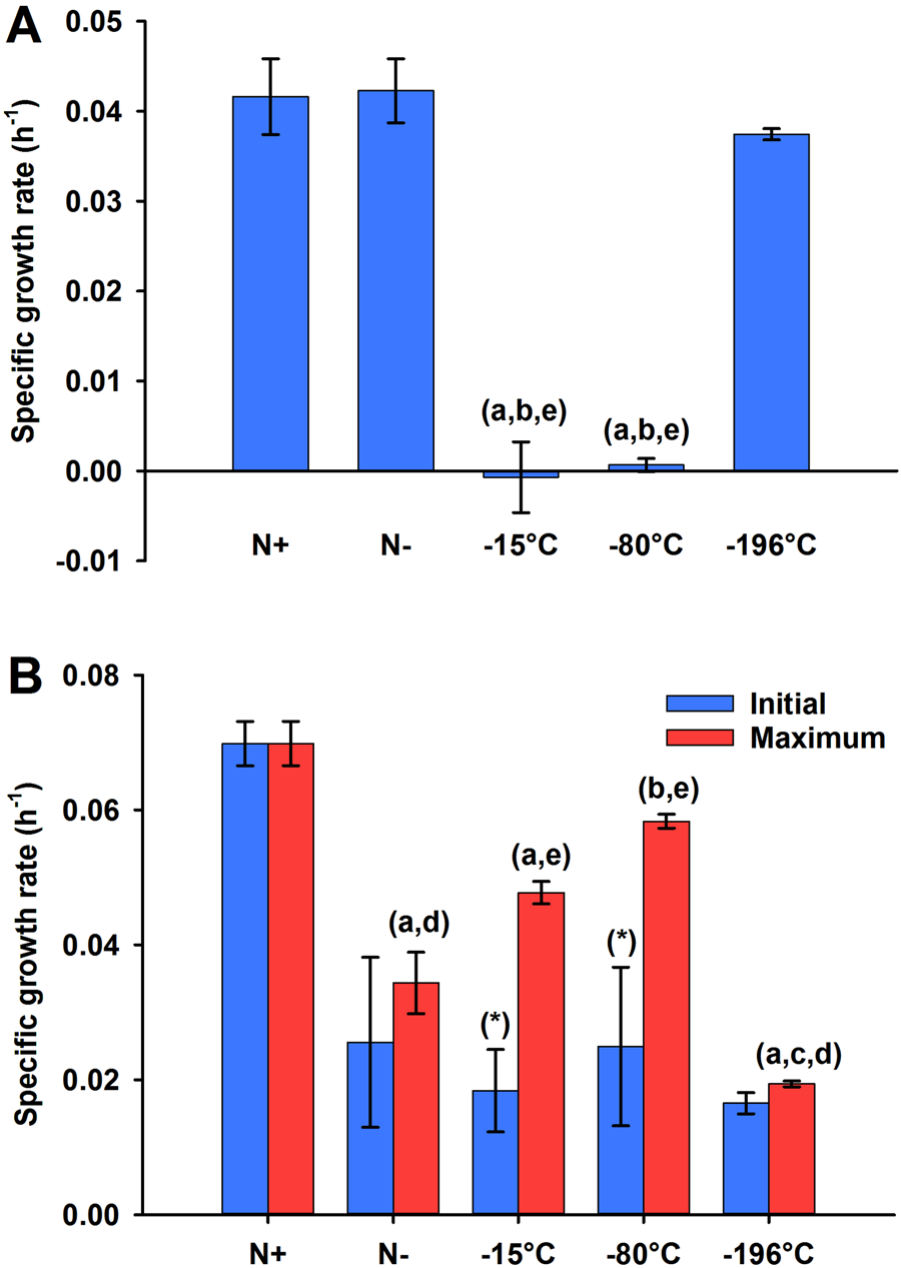
Specific growth rates (h^−1^) observed during 0–24 h in Phase 1 (**A**), and at “Initial” (0–4 h) and “Maximum” (over 0–72 h) in Phase 2 (**B**). The specific growth rates were measured as μ = ln(*X*_*t*_/*X*_0_)/(t_t_ − t_0_), *X*_*t*_ and *X*_0_ being the biomass concentrations (as OD_595nm_) at time points t hours (t_t_) and 0 hours (t_0_), respectively. Mean (±standard error) values of three biological replicates are plotted. Analysis of the variance followed by the Bonferroni post hoc test (*p*-value < 0.05) was carried out to estimate the significance of the differences between the treatments (i.e., (a) significant difference when compared with Control N replete, (b) significant difference when compared with Control N free, (c) significant difference when compared with −15 °C treated populations, (d) significant difference when compared with −80 °C treated populations and (e) significant difference when compared with −196 °C treated populations). In (**B**) the ANOVA between treatments was carried out for the maximum growth rates and between the “Initial” and the “Maximum” growth rates within the same treatment, (*) meaning significant difference between the two types of rate for each treatment.

The concentration of dissolved inorganic nitrogen (DIN) and phosphorous (DIP) in the culture media was measured in all the treatments. This showed that following the removal of nitrogen from the media at the beginning of Phase 2, no inorganic nitrogen was detected in the media (up to 32 h for the case of −15 °C and −80 °C) and that all the cryopreserved treatments continued to be nitrogen starved during the duration of the physiological experiment (Fig. 2A). −15 °C and−80 °C cryopreserved strains showed a slight increase in the DIN from 32 h onwards, perhaps, as a result of remineralisation of organic nitrogen released into the media by these cultures. The DIP was consumed in the first 4 hours of nitrogen starvation in all treatments (Fig. 2B). After recovery from the cryopreservation treatments, both −15 °C and −80 °C stored cultures were slower in picking up growth under the replete nutrient conditions of Phase 1 (0–24 h) when compared to the −196 °C treatment (*p*-value < 0.001) (Fig. 3A). This was noted despite equivalent prior acclimatisation (Fig. 1). As expected, the initial growth rates (0–4 h) for Phase 2 of all the cultures under nitrogen starvation (the Control N free and the three cryopreserved cultures) were slower than the initial growth rate observed for the Control N replete, suggesting the requirement for a certain time to acclimatise the cultures to changes in nutrient concentrations after the transfer (Fig. 3B). However, while both the Control N free and the −196 °C cultures showed maximum growth rates (over the period of 72 h of cultivation) not significantly different to the initial rates and significantly slower than the Control N replete (p-value < 0.001), −15 °C and −80 °C stored cultures seemed to grow better than expected for the nitrogen starved treatment. Overall, the absence of nitrogen in the medium had a detrimental effect on the growth of *C. vulgaris* during the duration of the physiological experiment (72 h), with Control N free and −196 °C treatments arresting their growth at 24 h (Supplementary Fig. S1A).

**Figure 4:**
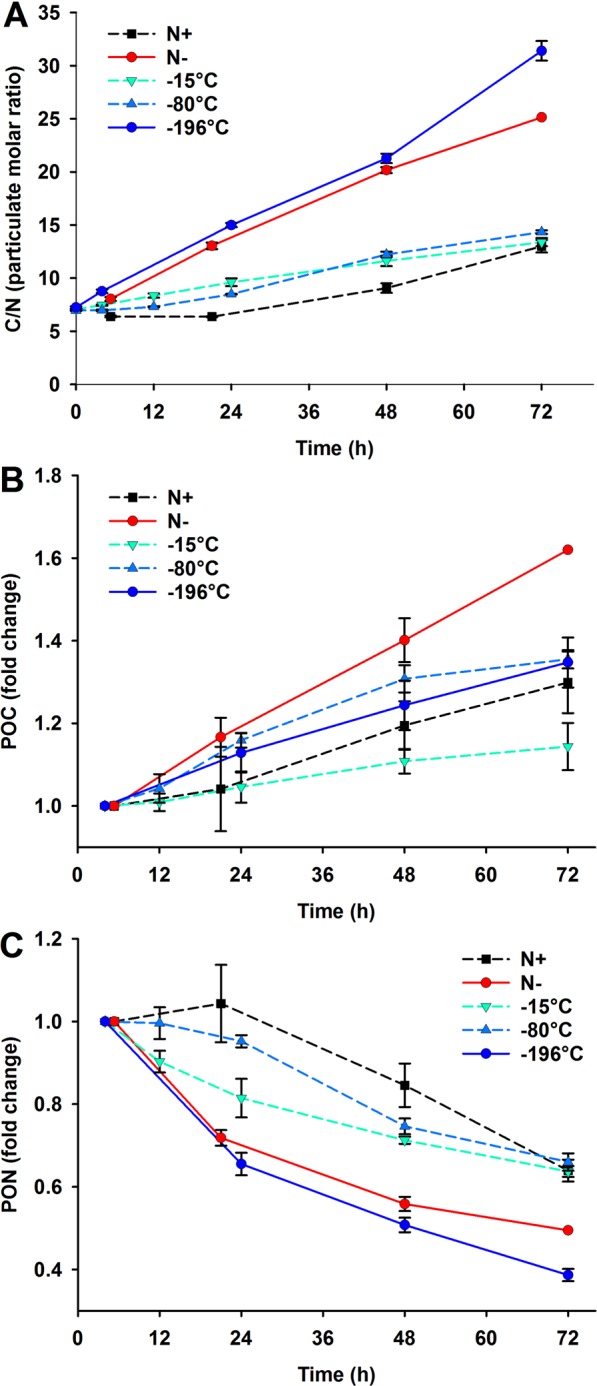
Element composition of the *C. vulgaris* −15 °C treated (inverted turquoise triangle and dashed line), −80 °C treated (pale blue triangle and dashed line) and −196 °C treated (dark blue circle and straight line) populations and of treatments prior to cryopreservation under nitrogen free conditions (Control N free, red circle and straight line) and nitrogen replete conditions (Control N replete, black square and dashed line). Mean (±standard error) of three biological replicates of (**A**) C/N molar ratio (derived from the Particulate Organic Carbon (POC) and the Particulate Organic Nitrogen (PON) reported in the supplementary Figure S1), (**B**) fold change in POC relative to initial time point measured (5 h), and (**C**) fold change in PON relative to initial time point measured (5 h).

In our study, the carbon to nitrogen (C/N) ratio was measured as a proxy to indicate the degree of nitrogen limitation within the algal cells. The C/N molar ratio rapidly increased to values higher than 10 at 24 h in the −196 °C cryopreserved populations and in the Control N free treatment. For the −15 °C and −80 °C stored populations, the increase was slower reaching a C/N of 10 at 48 h (Fig. 4A). The Control N replete *C. vulgaris* population did not show signs of intracellular nitrogen limitation until the end of the experimentation when the nitrogen in the medium was exhausted. The intracellular particulate organic carbon (POC) increased by 20–60% of the initial value over 72 h in all the treatments, except the −15 °C stored culture, where the increase was minimal at around 10% (Fig. 4B). A concomitant decrease in intracellular particulate organic nitrogen (PON) was noticed in all the treatments, with a dramatic drop to 60% in the first 24 h and a further drop to 40% of the initial value in 72 h in the −196 °C cryopreserved and 50% in Control N free populations, contrasting with the more gentle drop in the −15 °C and −80 °C stored cultures that reached values that were 60% of the initial values at 72 h, more similar to that noticed with the Control N replete condition (Fig. 4C). The cultures stored at −15 °C and −80 °C thus seemed to have a better ability to retain the intracellular nitrogen than the −196 °C cryopreserved and Control N free populations, showing both higher biomass productivity (indicated by having ODs 50% higher from 48 h onwards - Supplementary Fig. S1A) and higher PON levels which were between >50% higher from 24 h until the end of the experiment. No noticeable increase of DIN in −196 °C stored and Control N free cultures was detected however (−15 °C and −80 °C stored cultures showed levels of DIN in the media of up to 25–26 uM in the latter part of Phase 2: Fig. 2A).

**Figure 5:**
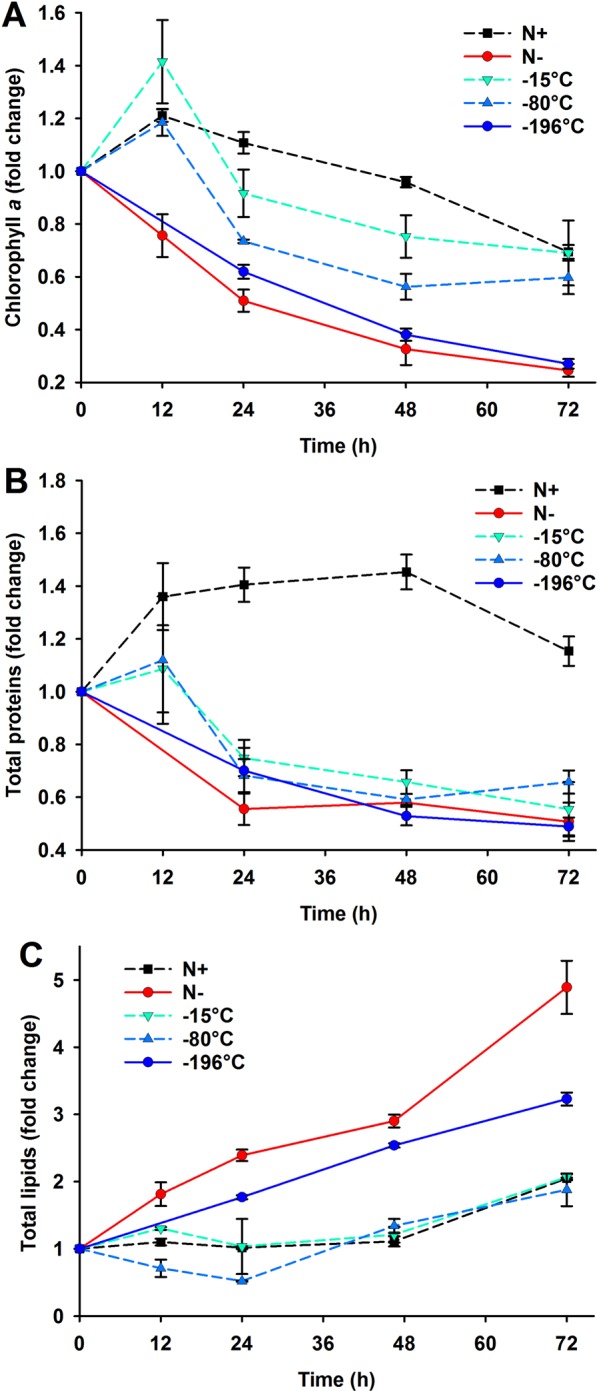
Fold change with respect to initial measurements (0 h) of the biochemical composition for the cryopreserved populations under nitrogen starved conditions. Mean (±standard error) fold change of three biological replicates are shown for (**A**) Chlorophyll *a*; (**B**) Total proteins and (**C**) Total lipids for *C. vulgaris* −15 °C treated (inverted turquoise triangle and dashed line), −80 °C treated (pale blue triangle and dashed line) and −196 °C treated (dark blue circle and straight line) populations and prior to cryopreservation treatments under nitrogen free conditions (Control N free, red circle and straight line) and nitrogen replete conditions (Control N replete, black square and dashed line).

The −15 °C and −80 °C stored cultures also presented differences in their biochemical composition with phenotypes that were between an intracellular nitrogen limited and replete scenario, at least during the first 48 h of the nitrogen starvation. For instance, the −196 °C stored culture was observed to have a decrease in the chlorophyll *a* and total protein similar to that in the Control N free culture (Fig. 5A,B), which contrasted with the relatively unchanged concentrations in the Control N replete condition. Conversely, the −15 °C and −80 °C stored cultures had slower decreasing trends. Similar behaviour was observed with respect to lipids (Fig. 5C). Both Control N free and −196 °C stored cultures presented a steady increase in the lipid content from the start of the nitrogen starvation, although differences in the degree of accumulation were observed (5-fold and 3.5-fold, respectively). Finally, the −15 °C and −80 °C stored cultures clearly had their ability to accumulate lipids compromised by the cryopreservation storage temperatures employed, as their trends were similar to the changes observed for the Control N replete treatment.

## Discussion

Whilst the use of suboptimal environmental conditions including incubation temperature and illumination (for low growth) is the standard approach in maintaining live algal cultures^9^, it is considered inadequate in the context of biotechnologically exploited materials in terms of both strain security and ensuring culture “performance”^20,30^. Where effective methodologies have been developed, low temperature storage, specifically ultra-low temperature cryopreservation, is accepted as the optimal approach^8,20,30^. The tolerance of biological materials to cryopreservation depends upon the ability of the material/organism being cooled to overcome or avoid cryo-injury induced by physical and chemical changes associated with freezing of the surrounding medium and the material/organisms themselves. Mazur^31^ proposed that two factors were involved namely: ice formation and colligative damage due to excessive concentrations of solutes as ice is formed. There is a considerable body of work exploring both the physics of cryopreservation and its effects on biological systems, for comprehensive reviews see Taylor (1987)^32^ and Muldrew *et al*.^33^, respectively. From a practical perspective, for many biological materials (from viruses to mammalian embryos) standard methodologies applicable to a range of organisms/materials have been developed^34,35^. However, for algae, as for other groups of organisms, it is clear that there is no universally applicable method, although “standard” protocols, applicable for a variety of cyanobacteria^19^, microalgae^36^ and macroalgae^37^, have been published. Irrespective of the methodological approach, stability of the stored samples is of paramount importance. In theory, where storage temperatures are sufficiently low to prevent molecular motion, cryogenically stored samples should be effectively inert. Thus, the −196 °C preserved cells are in an “arrested” state and long-term storage should result in little or no loss of viability. The key to this stability is the maintenance of samples in a glassy (vitrified) state, where neither solute/salt nor ice-induced injuries can occur. To achieve this, cryopreserved samples need to be stored at temperatures less than the critical glass transition temperature (*T*_g_), which for pure water has been reported as being between −130 °C and −132 °C^38^. Wowk (2010)^39^ reported that *T*_g_ is relevant to the stability of cryopreserved samples in the totally vitrified state and in the frozen state, where cells survive in glasses between ice crystals. In the majority of samples that are thought of as frozen (i.e. with a large content of crystalline ice), rather than vitrified (i.e. water and other components form a glass) there are assumed to be regions within the sample where a ‘glassy’ matrix has been formed^40^.

In common with other biotechnological sectors, few algal biotechnologists have a significant level of cryobiological experience and are unaware of the potential implications of employing different storage temperatures. Furthermore, although sample stability is critical, an equally important consideration is that methods should be practical. This had led to a number of workers exploring the potential of employing domestic freezers^41,42^, as these are readily available in virtually all labs and production facilities. Furthermore, −80 °C storage is the standard approach for the conservation of bacterial cultures^27^ and has been successfully employed for a variety of eukaryotic organisms^8,34^. Irrespective of the protocols employed, the objectives of any cryopreservation procedure are twofold: firstly, to have viable material capable of regenerating a new culture, plant or animal and secondly, the revived material should regenerate as phenotypically and functionally “normal” i.e. stored revived materials must be “fit for purpose”. In this study, we systematically investigated the effects of cryopreservation and storage of a lipid producing alga *C. vulgaris* CCAP 211/21A. This alga was selected because it is the model strain on which a substantial body of research has been performed in our labs^29,43,44^ and we were confident of the stability of the alga under its standard maintenance regime. In addition, its relative *C. vulgaris* CCAP 211/11B, which is employed worldwide in ecotoxicology^45^, has been demonstrated to retain its genotypic stability for >40 years on being serially transferred under different cultivation regimes and under liquid nitrogen storage^10^. The storage temperatures were chosen because they reflect equipment availability and we selected a −15 °C, rather than a −20 °C, domestic freezer as we wanted to explore the effect of the highest sub-zero temperature that would ensure that the material, or its external medium, were frozen. It is worth noting that samples in marine-based algal media may supercool to <−12 °C before extracellular ice nucleation occurs^11^. The period of 4 months was chosen as this is the shortest duration of storage that is less labour intensive in comparison to routine serial transfer for this alga.

On storage at −15 °C, we observed an unacceptably rapid reduction in viability of *C. vulgaris* CCAP 211/21A to <1% and failure to revive some samples within one month’s storage. This is similar to an earlier report^46^, where green spores of the ferns *Osmuncla regalis* and *Equisetum ramosissimum* lost viability quickly, with unacceptably high levels of variability on storage at −25 °C over a 24-month storage period. In this study, storage at −80 °C was suboptimal with a 70% reduction in viability levels, although all replicates tested up to 4 months storage revived and regenerated actively growing cultures. It may be assumed that the reduction in viability was due to damage to algal cells associated with ice crystal development and other physico-chemical changes in the ice matrix at this relatively high sub-zero storage temperature. For some eukaryotic organisms, −80 °C storage has proven to be satisfactory such as, for example, dried spores of *O. regalis* and *E. ramosissimum* which maintained high viability, rapid germination and normal growth over 24 months storage^46^. Furthermore, there are published examples of successful short-term (weeks-months) storage of eukaryotic algae at −80 °C^47,48^. As might be anticipated, based on cryobiological theory, materials held at −196 °C showed no reduction in viability over the four months storage. Current cryobiological understanding is that cells capable of functional recovery exist in the vitreous state at ultra-low temperatures, even though they may be surrounded by a ‘wall’ of ice^40^. This explains why storage below the critical *T*_g_ (~ −130 °C) is crucial to ensure viability for both frozen and totally vitrified samples. In this study, the samples that were plunged into liquid nitrogen would have contained vitrified cells within a frozen matrix. Samples subsequently stored at −80 °C will have devitrified when transferred from liquid nitrogen to the −80 °C freezer, this in itself did not result in any reduction in viability levels. However, on prolonged storage intracellular ice formation and salt induced injuries occurred in many algal cells, with lower recovery levels observed on thawing of the cryopreserved samples.

When conducting cryopreservation of living materials, in addition to testing for the viability of the storage cultures, it is equally important to determine that the functionality of the revived material is not lost and therefore is “still fit for purpose”. This is a fundamental requirement in biotechnology where maintaining a consistent phenotype and functionality is critical to keep a consistent commercial exploitation. However, for most practitioners, the approach employed is typically empirical (often copying a method used for some other organism), and there is no attempt to provide an adequately qualified reference stock of material to serve the increasingly sophisticated analytical systems for characterizing the sample^49^. Thus, there is often a lack of assurance that culture recovered in the future will have retained the properties of the original cells. The use of suboptimal storage regimes may further compound this issue, but to date, we are unaware of any reports of this being systematically investigated for algae. Ryan *et al*.^50^ reported that storage at −196 °C rather than −20 °C was preferable for the conservation of insecticidal secondary metabolite production by the fungi *Metarhizium anisopliae* and *Fusarium oxysporum*. More recently, evidence of molecular changes was reported to 18 genes associated with stress physiology, on storage of Human Peripheral Blood Mononuclear Cells (PBMCs) at −80 °C, but no changes were observed in samples stored <−150 °C^51^. Furthermore, as noted for *C. vulgaris*, PBMC recovery and viability were better when the cells were stored ≤−150 °C as compared to −80 °C^51^.

In our study, we assessed the effect of the different cryopreservation storage temperatures on the physiology of *C. vulgaris*, with a specific focus on the changes observed in its response to nitrogen starvation. This environmental condition is widely used in the algae biotechnology field to stimulate lipid production required for the generation of biofuels^29,52^. To achieve that goal, a physiological experiment involving cultivation under conditions of nitrogen starvation in the medium was conducted on algal cultures; before (Control N free) and after the cryopreservation and storage at the different temperatures (−15 °C, −80 °C and −196 °C), and any changes in the biochemical composition observed were compared to the growth of the same alga in the presence of nitrogen (Control N replete). The physiological responses to the nitrogen starvation were very similar in both algal cultures before (Control N free) and after the cryopreservation with storage at −196 °C. For instance, compared to the Control N replete culture, maximal growth rates were significantly lower in both Control N free and −196 °C derived cultures, with their growth arrested at 24 h, and the molar C/N ratio, a proxy of the intracellular nitrogen limitation, increased to levels up to 25 by the end of the physiological experiment. In addition, the decrease of chlorophyll *a* and increase of lipid content trends were very similar for both Control N free and −196 °C stored cultures and very different to that observed for the Control N replete cultures. These results coincide with what has been described for this and other species of algae subjected to the stress of nitrogen starvation^53,54^, and further endorse cryopreservation and storage at −196 °C for the long-term maintenance of *C. vulgaris* without compromising its functionality.

Contrasting to the results observed for samples stored at −196 °C, both −15 °C and −80 °C derived cultures showed different physiological responses to nitrogen starvation when compared to the Control N free treatment, having phenotypes that were between an intracellular nitrogen limited and replete scenario. For instance, they seemed to be more productive than the −196 °C and Control N free cultures, their growth was not arrested until towards the end of the physiological experiment, and their C/N ratios increased very slowly over time. In addition, although they showed a decreasing trend in their chlorophyll *a* and total protein content, the lipid content was very similar to the Control N replete culture, indicating that lipid accumulation was not stimulated, at least for the duration of the experiment. Finally, they seemed to release nitrogen to the environment, an ability that has been reported for different microalgae species both in the nature and in culture. All these differences together with the presence of a lag in the growth during the Phase 1 suggest that a sub-population of *C. vulgaris* had been selected through the cryopreservation/storage procedures at −15 °C and −80 °C and that cells with higher biomass productivity could be emerging from a sub-population of viable cells. This sub-population presented a new physiological phenotype, no longer directly comparable to the original culture, which was able to maintain growth in the absence of nitrogen for a longer period of time. The fact that this new phenotype presented higher PON levels than the −196 °C and Control N free cultures also pointed towards its physiological differentiation, as it contrasts with the loss of intracellular nitrogen due to organic matter secretion that has been previously described for chlorophyceans^55^.

In conclusion, this paper has provided important evidence that storage at high sub-zero temperatures cannot guarantee the stability of either viability or function for the alga studied. Storage at −15 °C and −80 °C seemed to select for a sub-population of *C. vulgaris* with very different physiological properties (able to keep growing for longer periods in the absence of nitrogen) than the original isolate. This change in functionality has profound biotechnological implications for the conservation of algae and other biological resources for biotechnology. It is advised that for commercially viable isolates, in particular, that conservation/cryopreservation methodologies should be optimised and functionality validated before the approach is used as standard practice.

## Methods

### Culture and routine maintenance regime

*C. vulgaris* CCAP 211/21A obtained from the Culture Collection of Algae and Protozoa was routinely maintained by the aseptic transfer of a 5% (v/v) inoculum into a 50 mL tissue culture flask, with a vent closure permitting gas exchange, containing 30 mL f/2 medium prepared according to Guillard (1975)^56^. Flasks were incubated at an irradiance ca. 40 µmol photons m^−2^ s^−1^ (measured at lid height and provided by fluorescence tubes, L36W/865 Lumilux® Cool Daylight, Osram GmbH, Germany), with a 12 h:12 h light-dark photoperiod at ca. 15 °C.

### Experimental design

To study the effect of different cryopreservation protocols on the physiological responses of *C. vulgaris* to nitrogen starvation, the same experiment was performed on four occasions. This involved studying triplicate samples prior to cryopreservation, this acted as a control, or initial characterization of the physiological status of the strain; and then on populations/cultures recovered from three cryopreservation treatments (see below). The physiological experiment was carried out in two phases (Fig. 1). In Phase 1, *C. vulgaris* was cultivated in 1 M Tris buffered f/2 medium^56^ in three 1 L volume cultures stirred at 300 rpm and aerated with 1% CO_2_ mixed with air. Aeration was filtered through a 0.22 µm filter and supplied with a Duran sparger, pore size 3, at a rate of 0.01 L m^−1^. The cultures were incubated at room temperature under continuous light at 200 µmol photons m^−2^ s^−1^ provided by cool white LED stripes. Initial inoculum density was at an optical density at 595 nm (OD_595_) of 0.15 (equivalent to ca. 10^6^ cells/mL) and growth was monitored daily by measuring OD_595_. When OD_595_ was in the range 0.5–0.6, the 3 × 1 L cultures were mixed and concentrated by centrifugation at 3500 *g* for 5 min in an Eppendorf 5810 centrifuge before being re-suspended to an equivalent OD_595_ of 0.6 (±0.1), as triplicates, in 1 L of f/2 medium free of nitrogen, which marked the start of the experimental Phase 2. In Phase 2, the same culture conditions were maintained with the exception of increasing the light to 250 µmol photons m^−2^ s^−1^. A similar physiological experiment using a normal f/2 medium in the second phase was performed prior to cryopreservation, to assess the growth under replete nutrient concentrations. Samples for determining cellular biochemical and elemental composition were taken at different time points. In addition, culture biomass was determined by measuring the OD_595_.

### Cryopreservation experiments

After the first (control) physiological experiment, two 15 mL samples of *C. vulgaris* were sent to the Scottish Association of Marine Sciences to conduct the cryopreservation experiments. On receipt, these were incubated for 24 h under the standard maintenance conditions outlined above. They were then combined and then three aliquots were subjected to three cooling and storage regimes.

#### Controlled rate cooler (CRC) method

Samples (10 mL) were mixed with sterile cryo-protective additive (CPA) to give a final CPA concentration of 5% (v/v) methanol in the f/2 medium. Aliquots (1 mL) were dispensed into cryovials (Greiner Bio-One GmbH, Germany). These were then incubated at room temperature (ca. 20 °C) for 15 min prior to transfer to a Planer Kryo 360-3.3 (Planer plc, UK) programmable freezer and cooled from room temperature at −1 °C min^−1^ to −40 °C, held at −40 °C for a further 15 min and then plunged into a dewar of liquid nitrogen (LN2). The samples were then transferred to the CCAP Cryostore and held in liquid-phase nitrogen (−196 °C).

#### Passive freezer method

Cryoprotectant solutions and cultures were prepared as outlined above. The Passive freezer unit, Mr. Frosty^TM^ (Nalgene NUNC International, USA) was prepared as per the manufacturer’s instructions, with the addition of 250 mL of isopropanol, then placed in a refrigerator overnight to equilibrate at 4 °C. Cryovials containing cryoprotectant-treated algae were transferred into the Mr. Frosty and the unit sealed and placed in a −80 °C freezer. This was then held in the freezer for 1.5 h, the frozen samples were then plunged into LN2, and then transferred back to the −80 °C freezer for storage.

#### Domestic freezer method

Cryoprotectant solutions and cultures were prepared as outlined above and 1 mL aliquots aseptically transferred into pre-labelled cryovials. The vials were then transferred directly to a domestic freezer at −15 °C.

#### Thawing and culture recovery

After storage, the vials were rapidly transferred to a 37 °C water bath and placed in floating vial holders. As soon as all ice had melted, 1 mL from each vial was aseptically transferred into a 50 mL tissue culture flask containing 25 mL of f/2 medium. The cultures were allowed to recover in complete darkness for 24 h and then incubated at 20 °C, under a 12 h:12 h light-dark photoperiod at 20 μmol photons m^2^ s^−1^.

#### Viability assessment

Viability levels were assessed on the basis of capacity to grow in liquid culture and to form discrete colonies in agar, according to the method of Day and Deville (1995)^57^. In brief, aliquots (1 mL) of logarithmic dilutions in sterile medium diluted to result in 50–500 cells mL^−1^ were aseptically transferred to pre-labelled Petri dishes (50 mm diameter) to which approximately 2.5 mL of medium (f/2) containing agar (1% w/v) was dispensed. After gelation of the agar, the plates were sealed with Parafilm and incubated, inverted, under standard algal culture conditions (see above) for 4 weeks. This procedure was repeated for thawed samples after 24 h, 1 month and 4 months storage. After 4 weeks incubation the agar plates were examined under a dissecting microscope (x 20 magnification), the number of colonies counted and viability expressed as a percentage of the original cell density of the control sample.

Cultures recovered after 4 months storage were incubated under the standard maintenance regime until a dense culture was obtained. These were then returned to the University of Sheffield for further study. On receipt, they were aseptically transferred to 1 M Tris buffered f/2 medium where they were acclimated to the laboratory conditions by incubation at room temperature in 250 mL flasks aerated with 0.22 µm filtered moisture air at 200 µmol photons m^−2^ s^−1^ irradiance. The recovered samples were maintained under these conditions in an exponential growth phase for 16 (−15 °C and −80 °C treated) and 22 (−196 °C) passages (replacing the medium every 4–6 days) before repeating the physiological experiment described above.

### Determination of DIN and DIP in the medium

After centrifuging the samples for the determination of the biochemical composition (see details below), supernatants were frozen and stored at −20 °C for further analysis of the medium nutrient composition. DIN in the media was determined by measuring the absorbance at 220 nm as described in Collos *et al*.^58^. Soluble Reactive Phosphate in the media (DIP) was determined following the method of Strickland and Parsons’s method (1968)^59^.

### Determination of the biochemical and elemental composition

All chemicals and analytical reagents were HPLC grade (Sigma-Aldrich, U.K.) unless stated otherwise. Samples (5 mL) from each bottle were centrifuged at 3500 *g* for 5 min. Pellets were washed with phosphate buffer 0.01 M before being transferred to 2 mL Eppendorf tubes and centrifuged at 7000 *g* for 2 min. After removing the supernatant, pellets were stored at −80 °C before being freeze-dried. Pigment (Chlorophyll *a*) and total protein concentrations were determined in the 5 mL-equivalent pellets of dry biomass using a modification of the combined assay described in Chen *et al*.^60^. For determining the Particulate Organic Carbon (POC) and Organic Nitrogen (PON) inside the cells, 15 mL samples were centrifuged and pellets freeze-dried as described above, and known amounts of dry biomass analysed in an Elemental Analyzer (Flash 2000) using L-Isoleucine as a standard.

### Determination of Total lipids. 

All chemicals and analytical reagents were of HPLC grade (Sigma-Aldrich, Dorset, U.K.) unless stated otherwise. A 5 mL algal culture was harvested by centrifugation at 19,000 *g* for 3 min to which 300 μL of toluene and 300 µL of 0.5 M sodium methoxide were added, followed by incubation at 80 °C for 20 min. After cooling to room temperature, 300 µL of 10% boron tri-fluoride in methanol was added and the mixture incubated at 80 °C for 20 min. After cooling to room temperature, 300 µL water and 600 µL of hexane were added. The mixture was vortexed for 1 min and centrifuged at 18,000 *g* at 4 °C for 10 min. The organic phase was recovered, measured and evaporated to dryness under inert nitrogen gas. The dried fatty acid methyl esters (FAMEs) were reconstituted in 80 μL hexane prior to identification and quantification as total lipids, as described elsewhere^61^.

### Statistical analysis

The statistical differences between the treatments for the physiological parameters determined were analysed by performing an analysis of the variance (ANOVA) followed by the Bonferroni t-test using the SigmaPlot software version 13.

## Supplementary information

Figure S1

## Data Availability

All data generated or analysed during this study are included in this published article (and its Supplementary Information files).

## References

[1] McWilliams, A. *Microbial Products: Technologies, Applications and Global Markets*. *BccResearch* (2015).

[2] Apt KE, Behrens PW (1999). Commercial developments in microalgal biotechnology. J. Phycol..

[3] Borowitzka MA (1999). Commercial production of microalgae: ponds, tanks, tubes and fermenters. J. Biotechnol..

[4] Cardozo KHM (2007). Metabolites from algae with economical impact. Comp. Biochem. Physiol. Part C Toxicol. Pharmacol..

[5] Muller-Feuga A (2000). The role of microalgae in aquaculture: situation and trends. J. Appl. Phycol..

[6] Becker EW (2007). Micro-algae as a source of protein. Biotechnol. Adv..

[7] Wijffels RH, Barbosa MJ (2010). An outlook on microalgal biofuels. Science (80-.)..

[8] Kirsop, B. E. & Doyle, A. Maintenance of Microorganisms and Cultured Cells: A Manual of Laboratory *Methods*. (Academic Press, 1991).

[9] Lorenz, M., Friedl, T. & Day, J. G. In *Algal Culturing Techniques* (ed. Anderson, R. A.) 145–155 (Elsevier Academic Press, 2005).

[10] Müller J, Friedl T, Hepperle D, Lorenz M, Day JG (2005). Distinction between multiple isolates of *Chlorella vulgaris* (Chlorophyta, Trebouxiophyceae) and testing for conspecificity using amplified fragment length polymorphism and its RDNA sequences. J. Phycol..

[11] Day JG, Fleck RA (2015). Cryo-injury in algae and the implications this has to the conservation of micro-algae. Microalgae Biotechnol..

[12] Leeson, E. A., Cann, J. P. & Morris, G. J. In *Maintenance of Microorganisms* (eds Kirsop, B. E. & Snell, J. J. S.) 131–160 (Academic Press, 1984).

[13] Cameron RE (1962). Species of *Nostoc Vaucher* Occurring in the Sonoran Desert in Arizona. Trans. Am. Microsc. Soc..

[14] Day, J. G., Priestley, I. M. & Codd, G. A. Storage, recovery and photosynthetic activities of immobilised algae. In *Plant and animal cells: process possibilities* (eds Webb, C. & Mavituna, F.) 257–261 (Chichester, West Sussex, 1987).

[15] Tindall, B. J. In *Cryopreservation and Freeze-Drying Protocols* (eds Day, J. G. & Stacey, G. N.) 368, 73–97 (Humana Press, 2007).

[16] Ryan, M. J. & Smith, D. In *Cryopreservation and Freeze-Drying Protocols* (eds Day, J. G. & Stacey, G. N.) 368, 127–140 (Humana Press, 2007).

[17] McGrath MS, Daggett P-M, Dilworth S (1978). Freeze-drying of algae: Chlorophyta and Chrysophyta. J. Phycol..

[18] Holm-Hansen, O. In Handbook of Phycological Methods: Culture Methods and Growth Measurements (ed. Stein, J. R.) 173–205 (Cambridge University Press, 1973).

[19] Day, J. G. In *Cyanobacteria: An Economic Perspective* (eds Sharma, N. K., Rai, A. K. & Stal, L. J.) 319–327 (John Wiley & Sons, Ltd, 2013).

[20] OECD. *OECD Best Practice Guidelines for Biological Resource Centres*. (2007).

[21] Day JG, Watanabe MM, Morris GJ, Fleck RA, McLellan MR (1997). Long-term viability of preserved eukaryotic algae. J. Appl. Phycol..

[22] Hédoin H (2006). *Porphyridium cruentum* A-408 and *Planktothrix* A-404 retain their capacity to produce biotechnologically exploitable metabolites after cryopreservation. J. Appl. Phycol..

[23] Hipkin R, Day JG, Rad-Menéndez C, Mock T (2014). The first evidence for genotypic stability in a cryopreserved transgenic diatom. J. Appl. Phycol..

[24] Wood SA (2008). Maintenance of cyanotoxin production by cryopreserved cyanobacteria in the New Zealand culture collection. New Zeal. J. Mar. Freshw. Res..

[25] Grout B, Morris J, McLellan M (1990). Cryopreservation and the maintenance of cell lines. Trends Biotechnol..

[26] Walters C, Wheeler L, Stanwood PC (2004). Longevity of cryogenically stored seeds. Cryobiology.

[27] Dando, T. R. & Bousfield, I. J. In *Maintenance of Microorganisms and Cultured Cells: A Manual of Laboratory Methods* (eds Kirsop, B. E. & Doyle, A.) 57–64 (Academic Press, 1991).

[28] Snell, J. J. S. & Kirsop, B. E. In *Maintenance of Microorganisms: A Manual of Laboratory Methods* (eds Kirsop, B. E. & Doyle, A.) 1–4 (Academic Press, 1984).

[29] Slocombe SP (2015). Unlocking nature’s treasure-chest: screening for oleaginous algae. Sci. Rep..

[30] Kirsop, B. E. & Hawksworth, D. L. The biodiversity of microorganisms and the role of microbial resource centres. *World Federation for Culture Collections* (1994).

[31] Mazur P (1965). The role of cell membranes in the freezing of yeast and other single cells. Ann. N. Y. Acad. Sci..

[32] Taylor, M. J. In *The Effects of Low Temperatures on Biological Systems*. (eds Grout, B. W. W. & Morris, G. J.) 3–71 (Edward Arnold, 1987).

[33] Muldrew, K., Acker, J. P., Elliott, J. A. W. & McGann, L. E. In *Life in the Frozen State* (eds. Fuller, B., Lane, N. & Benson, E.) 67–108 (CRC Press, 2004).

[34] Day, J. G. & Stacey, G. N. *Cryopreservation and Freeze-Drying Protocols*. (Humana Press, 2007).

[35] Wolkers, W. F. & Oldenhof, H. *Cryopreservation and Freeze-Drying Protocols*. **1257** (Springer, 2015).

[36] Day, J. G. & Brand, J. J. In *Algal Culturing Techniques* (ed. Anderson, R. A.) 165–187 (Elsevier Academic Press, 2005).

[37] Day, J. G. In *Protocols for Macroalgae Research* (eds Charrier, B., Wichard, T. & Reddy, C. R. K.) 496 (CRC Press, 2018).

[38] Benson EE (2008). Cryopreservation of Phytodiversity: A Critical Appraisal of Theory & Practice. CRC. Crit. Rev. Plant Sci..

[39] Wowk B (2010). Thermodynamic aspects of vitrification. Cryobiology.

[40] Benson EE, Betsou F, Fuller BJ, Harding K, Kofanova O (2013). Translating cryobiology principles into trans-disciplinary storage guidelines for biorepositories and biobanks: A concept paper. Cryo Letters.

[41] Gao G, Clare AS, Rose C, Caldwell GS (2017). Non-cryogenic preservation of thalli, germlings, and gametes of the green seaweed *Ulva rigida*. Aquaculture.

[42] Zhuang Y, Gong X, Zhang W, Gao W (2015). Cryopreservation of filaments of *Scytosiphon lomentaria* by vitrification. J. Appl. Phycol..

[43] Slocombe SP, Zhang Q, Black KD, Day JG, Stanley MS (2013). Comparison of screening methods for high-throughput determination of oil yields in micro-algal biofuel strains. J. Appl. Phycol..

[44] Slocombe SP, Ross M, Thomas N, McNeill S, Stanley MS (2013). A rapid and general method for measurement of protein in micro-algal biomass. Bioresour. Technol..

[45] OECD. *Manual for Investigation of HPV Chemicals: ANNEX 1: OECD Test Guidelines for Studies Included in the SIDS*. (1984).

[46] Ballesteros D, Estrelles E, Walters C, Ibars AM (2011). Effect of storage temperature on green spore longevity for the ferns *Equisetum ramosissimum and Osmunda regalis*. Cryo Letters.

[47] Benhra A, Ferard JF, Vasseur P (1994). Factorial design to optimize the viability of the alga *Scenedesmus subspicatus after cryopreservation*. Cryo Letters.

[48] Holm-Hansen O (1963). Viability of Blue-Green and Green Algae after Freezing. Physiol. Plant..

[49] Stacey GN, Day JG (2014). Putting cells to sleep for future science. Nat. Biotechnol..

[50] Ryan MJ, Smith D, Bridge PD, Jeffries P (2003). The relationship between fungal preservation method and secondary metabolite production in Metarhizium anisopliae and *Fusarium oxysporum*. World J. Microbiol. Biotechnol..

[51] Yang J (2016). The effects of storage temperature on PBMC gene expression. BMC Immunol..

[52] Rodolfi L (2009). Microalgae for oil: Strain selection, induction of lipid synthesis and outdoor mass cultivation in a low-cost photobioreactor. Biotechnol. Bioeng..

[53] Longworth J, Wu D, Huete-Ortega M, Wright PC, Vaidyanathan S (2016). Proteome response of *Phaeodactylum tricornutum*, during lipid accumulation induced by nitrogen depletion. Algal Res..

[54] Schmollinger S (2014). Nitrogen-Sparing Mechanisms in Chlamydomonas Affect the Transcriptome, the Proteome, and Photosynthetic Metabolism. Plant Cell.

[55] Hulatt CJ, Thomas DN (2010). Dissolved organic matter (DOM) in microalgal photobioreactors: A potential loss in solar energy conversion?. Bioresour. Technol..

[56] Guillard, R. R. L. In *Culture of Marine Invertebrate Animals* (eds Smith, W. L. & Chanley, M. H.) 29–60 (Plenum Press, 1975).

[57] Day, J. G. & DeVille, M. M. In *Cryopreservation and Freeze-Drying Protocols* (eds Day, J. G. & McLellan, M. R.) 81–90 (Humana Press, 1995). 10.1385/0-89603-296-5:1 7647848

[58] Collos Y (1999). An optical method for the rapid measurement of micromolar concentrations of nitrate in marine phytoplankton cultures. J. Appl. Phycol..

[59] Strickland, J. D. H. & Parsons, T. R. A manual of sea water analysis. *Bull. Fish. Res. Board Canada* **167** (1968).

[60] Chen Y, Vaidyanathan S (2013). Simultaneous assay of pigments, carbohydrates, proteins and lipids in microalgae. Anal. Chim. Acta.

[61] Kapoore, R. V. Mass spectrometry based hyphenated techniques for microalgal and mammalian metabolomics. (University of Sheffield, 2014).

