# Supplementary material for: Effects of cryopreservation on viability and functional stability of an industrially relevant alga
Source: Sci Rep. 2019 Feb 14;9:2093. doi: 10.1038/s41598-019-38588-6 (PMC6376116; doi:10.1038/s41598-019-38588-6)
Supplement: Supplementary file 1 — Figure S1 [file 41598_2019_38588_MOESM1_ESM.pdf]

Effects of cryopreservation on viability and functional stability of an industrially relevant alga

Rahul Vijay Kapoore[1§], María Huete-Ortega[1,3§], John G. Day[2§], Katarzyna Okurowska[1], Stephen P. Slocombe[2], Michele S. Stanley[2] and Seetharaman Vaidyanathan[1*]

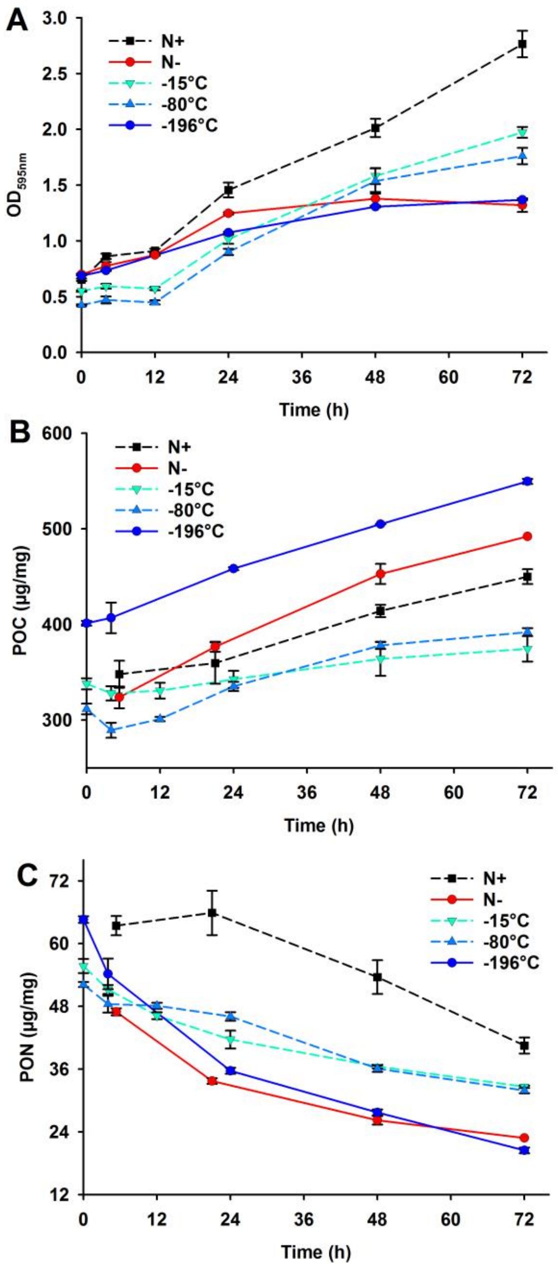

Supplementary Figure S1. Mean ± standard error of A) optical density, B) particulate organic carbon (POC) and C) particulate organic nitrogen (PON) of the *C. vulgaris* in Phase 2 growth. -15°C treated (inverted turquoise triangle and dashed line), -80°C treated (pale blue triangle and dashed line) and -196°C treated (dark blue circle and straight line) populations are shown along with controls before cryopreservation treatments under nitrogen free conditions (N-, red circle and straight line) and nitrogen replete conditions (N+, black square and dashed line).
